# Supplementary material for: Association between non-alcoholic fatty liver disease and arterial stiffness measured by brachial-ankle pulse wave velocity: a cross-sectional population study
Source: PeerJ. 2025 May 19;13:e19405. doi: 10.7717/peerj.19405 (PMC12097236; doi:10.7717/peerj.19405)
Supplement: Supplemental Information 1 — Multiple logistic regression was used to analyze the independent association of NAFLD and various confounders related to cardiovascular disease and metabolism with AS. Abbreviation: BMI, body mass index; TC, total cholesterol; TG, triglycerides; LDL, low-density lipoprotein; HDL, high-density lipoprotein; UA, uric acid; FBG, fasting blood glucose; NAFLD, non-alcoholic fatty liver disease. [file peerj-13-19405-s001.docx]

**Table S1：**

**Association Among AS and Clinical or Biochemical Variable**

| **Characteristics** | **OR** | **95%CL** | ***P* value** |
| --- | --- | --- | --- |
| Male | 1.374 | 1.165-1.619 | <0.001 |
| Age | 1.114 | 1.105-1.123 | <0.001 |
| BMI | 0.995 | 0.968-1.023 | 0.724 |
| High TC | 1.273 | 1.043-1.553 | 0.017 |
| High UA | 1.469 | 1.127-1.916 | 0.004 |
| High LDL | 0.991 | 0.749-1.107 | 0.348 |
| High TG | 1.365 | 1.161-1.604 | <0.001 |
| HDLC | 1.545 | 1.060-2.253 | 0.024 |
| Hypertension | 4.496 | 3.628-5.572 | 0.001 |
| High FBG | 1.703 | 1.268-2.287 | <0.001 |
| Current Smoking | 0.867 | 0.699-1.076 | 0.195 |
| Drinking | 1.003 | 0.763-1.318 | 0.985 |
| Exercise | 0.339 | 0.264-0.435 | <0.001 |
| NAFLD | 1.206 | 1.021-1.423 | 0.027 |

Multiple logistic regression was used to analyze the independent association of NAFLD and various confounders related to cardiovascular disease and metabolism with AS.

Abbreviation: BMI, body mass index; TC, total cholesterol; TG, triglycerides; LDL, low-density

lipoprotein; HDL: high-density lipoprotein; UA, uric acid; FBG, fasting blood glucose; NAFLD, non-alcoholic fatty liver disease.
