# Supplementary material for: Association between non-alcoholic fatty liver disease and arterial stiffness measured by brachial-ankle pulse wave velocity: a cross-sectional population study
Source: PeerJ. 2025 May 19;13:e19405. doi: 10.7717/peerj.19405 (PMC12097236; doi:10.7717/peerj.19405)
Supplement: Supplemental Information 2 — B*: Unstandardized β. Model 1 was adjusted for gender, age, BMI, smoking, drinking, and exercise; Model 2 further adjusted NAFLD based on Model 1; Model 3 further adjusted high TC, high TG, high UA, high FBG, and low HDL based on Model 2. [file peerj-13-19405-s002.docx]

**Table S2**

**Multiple linear regression model: Relationship between baPWV and multiple risk factors in the whole population**

| **Characters** | **Model 1** | | | **Model 2** | | |  | | **Model 3** | | | |
| --- | --- | --- | --- | --- | --- | --- | --- | --- | --- | --- | --- | --- |
|  | **β** | **VIF** | **P** | **β** | **VIF** | **P** | **β** | **B*** | | **VIF** | | **P** |
| Male | 0.105 | 1.167 | ＜0.001 | 0.099 | 1.174 | ＜0.001 | 0.091 | 55.875 | | | 1.209 | ＜0.001 |
| Age | 0.627 | 1.004 | ＜0.001 | 0.623 | 1.008 | ＜0.001 | 0.537 | 13.401 | | | 1.168 | ＜0.001 |
| BMI | 0.067 | 1.092 | ＜0.001 | 0.031 | 1.317 | 0.012 | -0.012 | -1.142 | | | 1.373 | 0.311 |
| smoking | -0.034 | 1.101 | 0.003 | -0.036 | 1.102 | 0.002 | -0.039 | -35.305 | | | 1.107 | ＜0.001 |
| drinking | -0.008 | 1.071 | 0.449 | -0.012 | 1.073 | 0.286 | -0.025 | -28.514 | | | 1.094 | 0.022 |
| exercise | -0.144 | 1.009 | ＜0.001 | -0.133 | 1.029 | ＜0.001 | -0.112 | -103.794 | | | 1.046 | ＜0.001 |
| NAFLD |  |  |  | 0.086 | 1.297 | ＜0.001 | 0.05 | 30.185 | | | 1.391 | ＜0.001 |
| Hypertension |  |  |  |  |  |  | 0.241 | 186.696 | | | 1.182 | ＜0.001 |
| High TC |  |  |  |  |  |  | 0.034 | 21.023 | | | 1.073 | 0.001 |
| High TG |  |  |  |  |  |  | 0.041 | 25.292 | | | 1.269 | ＜0.001 |
| High UA |  |  |  |  |  |  | 0.036 | 39.134 | | | 1.106 | 0.001 |
| High FBG |  |  |  |  |  |  | 0.043 | 50.124 | | | 1.066 | ＜0.001 |
| Low HDL |  |  |  |  |  |  | 0.004 | 6.484 | | | 1.048 | 0.683 |
| R² | 0.433 | | | 0.439 | | | 0.497 | | | | | |
| △R² | 0.433 | | | 0.006 | | | 0.058 | | | | | |
| F | 615.534 | | | 539.894 | | | 366.975 | | | | | |

B*: Unstandardized β. Model 1 was adjusted for gender, age, BMI, smoking, drinking, and exercise; Model 2 further adjusted NAFLD based on Model 1; Model 3 further adjusted high TC, high TG, high UA, high FBG, and low HDL based on Model 2
