# Supplementary material for: Association between non-alcoholic fatty liver disease and arterial stiffness measured by brachial-ankle pulse wave velocity: a cross-sectional population study
Source: PeerJ. 2025 May 19;13:e19405. doi: 10.7717/peerj.19405 (PMC12097236; doi:10.7717/peerj.19405)
Supplement: Supplemental Information 3 — Model 1 was adjusted for age, BMI, smoking, drinking, and exercise; Model 2 further adjusted NAFLD based on Model 1; Model 3 further adjusted high TC, high TG, high UA, high FBG, and low HDL based on Model 2. [file peerj-13-19405-s003.docx]

**Table S3**

**Multiple linear regression model: Relationship between baPWV and multiple risk factors in men**

| **Characters** | **Model 1** | | | **Model 2** | | | **Model 3** | | |
| --- | --- | --- | --- | --- | --- | --- | --- | --- | --- |
|  | **β** | **VIF** | **P** | **β** | **VIF** | **P** | **β** | **VIF** | **P** |
| Age | 0.589 | 1.007 | ＜0.001 | 0.587 | 1.008 | ＜0.001 | 0.508 | 1.168 | ＜0.001 |
| BMI | 0.035 | 1.019 | 0.016 | ＜0.001 | 1.244 | 0.978 | -0.044 | 1.309 | 0.004 |
| smoking | -0.044 | 1.031 | 0.002 | -0.046 | 1.031 | 0.001 | -0.048 | 1.037 | ＜0.001 |
| drinking | -0.011 | 1.038 | 0.455 | -0.014 | 1.041 | 0.325 | -0.030 | 1.063 | 0.028 |
| exercise | -0.137 | 1.012 | ＜0.001 | -0.126 | 1.034 | ＜0.001 | -0.104 | 1.058 | ＜0.001 |
| NAFLD |  |  |  | 0.081 | 1.263 | ＜0.001 | 0.052 | 1.343 | 0.001 |
| Hypertension |  |  |  |  |  |  | 0.241 | 1.161 | ＜0.001 |
| High TC |  |  |  |  |  |  | 0.030 | 1.070 | 0.033 |
| High TG |  |  |  |  |  |  | 0.035 | 1.252 | 0.019 |
| High UA |  |  |  |  |  |  | 0.056 | 1.083 | 0.001 |
| High FBG |  |  |  |  |  |  | 0.028 | 1.049 | 0.041 |
| Low HDL |  |  |  |  |  |  | 0.011 | 1.043 | 0.422 |
| R² | 0.369 | | | 0.374 | | | 0.432 | | |
| △R² | 0.370 | | | 0.005 | | | 0.058 | | |
| F | 370.407 | | | 315.453 | | | 200.572 | | |

Model 1 was adjusted for age, BMI, smoking, drinking, and exercise; Model 2 further adjusted NAFLD based on Model 1; Model 3 further adjusted high TC, high TG, high UA, high FBG, and low HDL based on Model 2
