# Supplementary material for: Association between non-alcoholic fatty liver disease and arterial stiffness measured by brachial-ankle pulse wave velocity: a cross-sectional population study
Source: PeerJ. 2025 May 19;13:e19405. doi: 10.7717/peerj.19405 (PMC12097236; doi:10.7717/peerj.19405)
Supplement: Supplemental Information 4 — Model 1 was adjusted for age, BMI, smoking, drinking, and exercise; Model 2 further adjusted NAFLD based on Model 1; Model 3 further adjusted high TC, high TG, high UA, high FBG, and low HDL based on Model 2. [file peerj-13-19405-s004.docx]

**Table S4**

**Multiple linear regression model: Relationship between baPWV and multiple risk factors in women**

| **Characters** | **Model 1** | | | **Model 2** | | | **Model 3** | | |
| --- | --- | --- | --- | --- | --- | --- | --- | --- | --- |
|  | **β** | **VIF** | **P** | **β** | **VIF** | **P** | **β** | **VIF** | **P** |
| Age | 0.685 | 1.037 | ＜0.001 | 0.672 | 1.064 | ＜0.001 | 0.569 | 1.262 | ＜0.001 |
| BMI | 0.082 | 1.038 | ＜0.001 | 0.05 | 1.203 | 0.006 | 0.016 | 1.233 | 0.351 |
| smoking | -0.007 | 1.000 | 0.682 | -0.005 | 1.001 | 0.764 | -0.012 | 1.011 | 0.438 |
| drinking | -0.013 | 1.002 | 0.445 | -0.016 | 1.003 | 0.342 | -0.02 | 1.009 | 0.197 |
| exercise | -0.156 | 1.003 | ＜0.001 | -0.145 | 1.019 | ＜0.001 | -0.127 | 1.028 | ＜0.001 |
| NAFLD |  |  |  | 0.088 | 1.241 | ＜0.001 | 0.042 | 1.339 | 0.020 |
| Hypertension |  |  |  |  |  |  | 0.251 | 1.242 | ＜0.001 |
| High TC |  |  |  |  |  |  | 0.030 | 1.098 | 0.066 |
| High TG |  |  |  |  |  |  | 0.031 | 1.194 | 0.066 |
| High UA |  |  |  |  |  |  | 0.014 | 1.025 | 0.387 |
| High FBG |  |  |  |  |  |  | 0.065 | 1.125 | ＜0.001 |
| Low HDL |  |  |  |  |  |  | -0.028 | 1.039 | 0.076 |
| R² | 0.527 | | | 0.533 | | | 0.598 | | |
| △R² | 0.528 | | | 0.006 | | | 0.066 | | |
| F | 377.014 | | | 321.987 | | | 209.778 | | |

Model 1 was adjusted for age, BMI, smoking, drinking, and exercise; Model 2 further adjusted NAFLD based on Model 1; Model 3 further adjusted high TC, high TG, high UA, high FBG, and low HDL based on Model 2
