# Supplementary material for: Association between non-alcoholic fatty liver disease and arterial stiffness measured by brachial-ankle pulse wave velocity: a cross-sectional population study
Source: PeerJ. 2025 May 19;13:e19405. doi: 10.7717/peerj.19405 (PMC12097236; doi:10.7717/peerj.19405)
Supplement: Supplemental Information 5 — Model 1 was adjusted for gender, age, BMI, smoking, drinking, and exercise; Model 2 further adjusted NAFLD based on Model 1; Model 3 further adjusted high TC, high TG, high UA, high FBG, and low HDL based on Model 2. [file peerj-13-19405-s005.docx]

**Table S5**

**Sensitivity analysis of multiple linear regression models: Relationship between baPWV and multiple risk factors in the whole population**

| **Characters** | **Model 1** | | | **Model 2** | | | **Model 3** | | |
| --- | --- | --- | --- | --- | --- | --- | --- | --- | --- |
|  | **β** | **VIF** | **P** | **β** | **VIF** | **P** | **β** | **VIF** | **P** |
| Male | 0.105 | 1.167 | ＜0.001 | 0.099 | 1.174 | ＜0.001 | 0.092 | 1.209 | ＜0.001 |
| Age | 0.613 | 1.004 | ＜0.001 | 0.608 | 1.009 | ＜0.001 | 0.527 | 1.145 | ＜0.001 |
| BMI | 0.071 | 1.090 | ＜0.001 | 0.040 | 1.300 | ＜0.001 | -0.009 | 1.361 | 0.434 |
| smoking | -0.035 | 1.102 | 0.002 | -0.037 | 1.103 | 0.001 | -0.038 | 1.107 | ＜0.001 |
| drinking | -0.013 | 1.074 | 0.231 | -0.016 | 1.076 | 0.150 | -0.025 | 1.093 | 0.017 |
| exercise | -0.142 | 1.010 | ＜0.001 | -0.133 | 1.028 | ＜0.001 | -0.111 | 1.045 | ＜0.001 |
| NAFLD |  |  |  | 0.077 | 1.281 | ＜0.001 | 0.050 | 1.364 | ＜0.001 |
| Hypertension |  |  |  |  |  |  | 0.252 | 1.157 | ＜0.001 |
| High TC |  |  |  |  |  |  | 0.036 | 1.064 | 0.001 |
| High TG |  |  |  |  |  |  | 0.040 | 1.258 | ＜0.001 |
| High UA |  |  |  |  |  |  | 0.030 | 1.099 | 0.004 |
| High FBG |  |  |  |  |  |  | 0.038 | 1.051 | ＜0.001 |
| Low HDL |  |  |  |  |  |  | 0.005 | 1.021 | 0.652 |
| R² | 0.417 | | | 0.422 | | | 0.484 | | |
| △R² | 0.417 | | | 0.005 | | | 0.063 | | |
| F | 608.128 | | | 40.295 | | | 103.015 | | |

Model 1 was adjusted for gender, age, BMI, smoking, drinking, and exercise; Model 2 further adjusted NAFLD based on Model 1; Model 3 further adjusted high TC, high TG, high UA, high FBG, and low HDL based on Model 2
