# Supplementary material for: Association between non-alcoholic fatty liver disease and arterial stiffness measured by brachial-ankle pulse wave velocity: a cross-sectional population study
Source: PeerJ. 2025 May 19;13:e19405. doi: 10.7717/peerj.19405 (PMC12097236; doi:10.7717/peerj.19405)
Supplement: Supplemental Information 6 — Model 1 was adjusted for gender, age, BMI, smoking, drinking, and exercise; Model 2 further adjusted NAFLD based on Model 1; Model 3 further adjusted high TC, high TG, high UA, high FBG, and low HDL based on Model 2. [file peerj-13-19405-s006.docx]

**Table S6**

**Sensitivity analysis of multiple linear regression models: Relationship between baPWV and multiple risk factors in men**

| **Characters** | **Model 1** | | | **Model 2** | | | **Model 3** | | |
| --- | --- | --- | --- | --- | --- | --- | --- | --- | --- |
|  | **β** | **VIF** | **P** | **β** | **VIF** | **P** | **β** | **VIF** | **P** |
| Age | 0.571 | 1.008 | ＜0.001 | 0.569 | 1.009 | ＜0.001 | 0.494 | 1.145 | ＜0.001 |
| BMI | 0.041 | 1.020 | 0.004 | 0.012 | 1.231 | 0.446 | -0.038 | 1.302 | 0.013 |
| smoking | -0.045 | 1.032 | 0.002 | -0.047 | 1.033 | ＜0.001 | -0.047 | 1.038 | ＜0.001 |
| drinking | -0.017 | 1.039 | 0.244 | -0.019 | 1.041 | 0.179 | -0.031 | 1.060 | 0.026 |
| exercise | -0.136 | 1.011 | ＜0.001 | -0.127 | 1.030 | ＜0.001 | -0.104 | 1.057 | ＜0.001 |
| NAFLD |  |  |  | 0.071 | 1.245 | ＜0.001 | 0.050 | 1.314 | 0.001 |
| Hypertension |  |  |  |  |  |  | 0.252 | 1.132 | ＜0.001 |
| High TC |  |  |  |  |  |  | 0.028 | 1.063 | 0.039 |
| High TG |  |  |  |  |  |  | 0.033 | 1.241 | 0.026 |
| High UA |  |  |  |  |  |  | 0.049 | 1.076 | ＜0.001 |
| High FBG |  |  |  |  |  |  | 0.026 | 1.037 | 0.055 |
| Low HDL |  |  |  |  |  |  | 0.010 | 1.027 | 0.450 |
| R² | 0.349 | | | 0.354 | | | 0.415 | | |
| △R² | 0.349 | | | 0.005 | | | 0.063 | | |
| F | 353.515 | | | 20.433 | | | 58.937 | | |

Model 1 was adjusted for gender, age, BMI, smoking, drinking, and exercise; Model 2 further adjusted NAFLD based on Model 1; Model 3 further adjusted high TC, high TG, high UA, high FBG, and low HDL based on Model 2
