# Supplementary material for: Association between non-alcoholic fatty liver disease and arterial stiffness measured by brachial-ankle pulse wave velocity: a cross-sectional population study
Source: PeerJ. 2025 May 19;13:e19405. doi: 10.7717/peerj.19405 (PMC12097236; doi:10.7717/peerj.19405)
Supplement: Supplemental Information 7 — Model 1 was adjusted for gender, age, BMI, smoking, drinking, and exercise; Model 2 further adjusted NAFLD based on Model 1; Model 3 further adjusted high TC, high TG, high UA, high FBG, and low HDL based on Model 2. [file peerj-13-19405-s007.docx]

**Table S7**

**Sensitivity analysis of multiple linear regression models: Relationship between baPWV and multiple risk factors in women**

| **Characters** | **Model 1** | | | **Model 2** | | | **Model 3** | | |
| --- | --- | --- | --- | --- | --- | --- | --- | --- | --- |
|  | **β** | **VIF** | **P** | **β** | **VIF** | **P** | **β** | **VIF** | **P** |
| Age | 0.678 | 1.038 | ＜0.001 | 0.666 | 1.066 | ＜0.001 | 0.565 | 1.241 | ＜0.001 |
| BMI | 0.079 | 1.039 | ＜0.001 | 0.051 | 1.193 | 0.004 | 0.008 | 1.225 | 0.617 |
| smoking | -0.007 | 1.000 | 0.681 | -0.005 | 1.001 | 0.752 | -0.011 | 1.010 | 0.457 |
| drinking | -0.014 | 1.001 | 0.404 | -0.016 | 1.003 | 0.314 | -0.020 | 1.008 | 0.177 |
| exercise | -0.152 | 1.004 | ＜0.001 | -0.143 | 1.020 | ＜0.001 | -0.123 | 1.029 | ＜0.001 |
| NAFLD |  |  |  | 0.080 | 1.230 | ＜0.001 | 0.043 | 1.328 | 0.014 |
| Hypertension |  |  |  |  |  |  | 0.258 | 1.224 | ＜0.001 |
| High TC |  |  |  |  |  |  | 0.033 | 1.085 | 0.035 |
| High TG |  |  |  |  |  |  | 0.031 | 1.187 | 0.064 |
| High UA |  |  |  |  |  |  | 0.010 | 1.026 | 0.512 |
| High FBG |  |  |  |  |  |  | 0.068 | 1.108 | ＜0.001 |
| Low HDL |  |  |  |  |  |  | -0.016 | 1.004 | 0.0298 |
| R² | 0.517 | | | 0.522 | | | 0.590 | | |
| △R² | 0.518 | | | 0.005 | | | 0.069 | | |
| F | 3385.534 | | | 19.528 | | | 550.271 | | |

Model 1 was adjusted for gender, age, BMI, smoking, drinking, and exercise; Model 2 further adjusted NAFLD based on Model 1; Model 3 further adjusted high TC, high TG, high UA, high FBG, and low HDL based on Model 2
