# Supplementary material for: Association between non-alcoholic fatty liver disease and arterial stiffness measured by brachial-ankle pulse wave velocity: a cross-sectional population study
Source: PeerJ. 2025 May 19;13:e19405. doi: 10.7717/peerj.19405 (PMC12097236; doi:10.7717/peerj.19405)
Supplement: Supplemental Information 8 — *:Gender subgroups in the NAFLD population. Group 1: “<35”; Group 2: “[35, 45)”; Group 3: “[45, 55)”; Group 4: “[55, 65)”; Group 5: “ ≥ 65”. [file peerj-13-19405-s008.docx]

**Table S8**

**Post-hoc multiple comparisons of ANOVA for age-stratified analysis**

|  | **Comparison** | **Mean Difference** | **SE** | **95%CI** | **P** |
| --- | --- | --- | --- | --- | --- |
| NAFLD | Group 1 vs. Group 2 | -79.44 | 23.01 | [-124.56, -34.31] | 0.001 |
|  | Group 1 vs. Group 3 | -162.92 | 21.68 | [-205.44, -120.41] | ＜0.001 |
|  | Group 1 vs. Group 4 | -313.25 | 23.08 | [-358.52, -267.99] | ＜0.001 |
|  | Group 1 vs. Group 5 | -681.42 | 26.44 | [-733.27, -629.57] | ＜0.001 |
|  | Group 2 vs. Group 3 | -83.48 | 14.53 | [-111.97, -54.99] | ＜0.001 |
|  | Group 2 vs. Group 4 | -233.81 | 16.55 | [-266.27, -201.36] | ＜0.001 |
|  | Group 2 vs. Group 5 | -601.98 | 20.98 | [-643.12, -560.84] | ＜0.001 |
|  | Group 3 vs. Group 4 | -150.33 | 14.64 | [-179.05, -121.61] | ＜0.001 |
|  | Group 3 vs. Group 5 | -518.50 | 19.51 | [-556.76, -480.23] | ＜0.001 |
|  | Group 4 vs. Group 5 | -368.16 | 21.06 | [-409.46, -326.86] | ＜0.001 |
| Non-NAFLD | Group 1 vs. Group 2 | -79.44 | 23.01 | [-124.56, -34.32] | ＜0.001 |
|  | Group 1 vs. Group 3 | -162.92 | 21.68 | [-205.44, -120.41] | ＜0.001 |
|  | Group 1 vs. Group 4 | -313.25 | 23.08 | [-358.52, -267.99] | ＜0.001 |
|  | Group 1 vs. Group 5 | -681.42 | 26.44 | [-733.27, -629.57] | ＜0.001 |
|  | Group 2 vs. Group 3 | -83.48 | 14.53 | [-111.97, -111.97] | ＜0.001 |
|  | Group 2 vs. Group 4 | -233.81 | 16.55 | [-266.27, -201.35] | ＜0.001 |
|  | Group 2 vs. Group 5 | -601.98 | 20.98 | [-643.12, -560.84] | ＜0.001 |
|  | Group 3 vs. Group 4 | -150.33 | 14.64 | [-179.05, -121.61] | ＜0.001 |
|  | Group 3 vs. Group 5 | -518.50 | 19.51 | [-556.76, -480.23] | ＜0.001 |
|  | Group 4 vs. Group 5 | -368.16 | 21.06 | [-409.46, -326.86] | ＜0.001 |
| Male * | Group 1 vs. Group 2 | -63.76 | 23.67 | [-110.19, -17.32 | ＜0.001 |
|  | Group 1 vs. Group 3 | -149.36 | 22.39 | [-193.29, -105.44] | ＜0.001 |
|  | Group 1 vs. Group 4 | -278.80 | 24.69 | [-327.24, -230.36] | ＜0.001 |
|  | Group 1 vs. Group 5 | -638.52 | 29.95 | [-697.26, -579.78] | ＜0.001 |
|  | Group 2 vs. Group 3 | -85.61 | 15.12 | [-115.26, -55.96] | ＜0.001 |
|  | Group 2 vs. Group 4 | -215.04 | 18.35 | [-251.04, -179.04] | ＜0.001 |
|  | Group 2 vs. Group 5 | -574.76 | 24.97 | [-623.76, -525.77] | ＜0.001 |
|  | Group 3 vs. Group 4 | -129.43 | 16.67 | [-162.14, -96.73] | ＜0.001 |
|  | Group 3 vs. Group 5 | -489.16 | 23.77 | [-535.78, -442.53] | ＜0.001 |
|  | Group 4 vs. Group 5 | -359.72 | 25.95 | [-410.62, -308.82] | ＜0.001 |
| Female* | Group 1 vs. Group 2 | -180.98 | 69.74 | [-318.08, -43.89] | ＜0.001 |
|  | Group 1 vs. Group 3 | -268.64 | 64.54 | [-395.50, -141.78] | ＜0.001 |
|  | Group 1 vs. Group 4 | -484.09 | 64.45 | [-610.78, -357.40] | ＜0.001 |
|  | Group 1 vs. Group 5 | -858.22 | 67.53 | [-990.96, -725.48] | ＜0.001 |
|  | Group 2 vs. Group 3 | -87.66 | 41.63 | [-169.50, -5.82] | ＜0.001 |
|  | Group 2 vs. Group 4 | -303.11 | 41.50 | [-384.68, -221.54] | ＜0.001 |
|  | Group 2 vs. Group 5 | -677.23 | 46.13 | [-767.91, -586.56] | ＜0.001 |
|  | Group 3 vs. Group 4 | -215.45 | 31.99 | [-278.33, -152.57] | ＜0.001 |
|  | Group 3 vs. Group 5 | -589.58 | 37.81 | [-663.89, -515.26] | ＜0.001 |
|  | Group 4 vs. Group 5 | -374.13 | 37.66 | [-448.15, -300.11] | ＜0.001 |

*: Gender subgroups in the NAFLD population.

Group 1: “< 35”; Group 2: “[35, 45)”; Group 3: “[45,55)”; Group 4: “[55, 65)”; Group 5: “≥65”.
