# Supplementary material for: Association between non-alcoholic fatty liver disease and arterial stiffness measured by brachial-ankle pulse wave velocity: a cross-sectional population study
Source: PeerJ. 2025 May 19;13:e19405. doi: 10.7717/peerj.19405 (PMC12097236; doi:10.7717/peerj.19405)
Supplement: Supplemental Information 9 — Model 1 adjusted for age, BMI, smoking, drinking, and exercise; Model 2 further adjusted NAFLD based on Model 1; Model 3 further adjusted high TC, high TG, high UA, high FBG, and low HDL on the basis of Model 2. [file peerj-13-19405-s009.docx]

**Table S9**

**Multiple linear regression model after score matching**

| **Characters** | **Model 1** | | | **Model 2** | | | **Model 3** | | |
| --- | --- | --- | --- | --- | --- | --- | --- | --- | --- |
|  | **β** | **VIF** | **P** | **β** | **VIF** | **P** | **β** | **VIF** | **P** |
| Male | 0.050 | 1.099 | ＜0.001 | 0.051 | 1.100 | 0.001 | 0.047 | 1.138 | 0.002 |
| Age | 0.630 | 1.041 | 0.002 | 0.63 | 1.041 | ＜0.001 | 0.535 | 1.245 | ＜0.001 |
| BMI | 0.000 | 1.025 | 0.996 | -0.003 | 1.030 | 0.852 | -0.025 | 1.045 | 0.088 |
| smoking | -0.015 | 1.081 | 0.337 | -0.016 | 1.081 | 0.303 | -0.026 | 1.090 | 0.083 |
| drinking | -0.016 | 1.062 | 0.308 | -0.017 | 1.062 | 0.287 | -0.031 | 1.086 | 0.041 |
| exercise | -0.089 | 1.008 | ＜0.001 | -0.089 | 1.008 | ＜0.001 | -0.079 | 1.014 | ＜0.001 |
| NAFLD |  |  |  | 0.042 | 1.006 | 0.005 | 0.040 | 1.008 | 0.006 |
| Hypertension |  |  |  |  |  |  | 0.254 | 1.170 | ＜0.001 |
| High TC |  |  |  |  |  |  | 0.026 | 1.055 | 0.076 |
| High TG |  |  |  |  |  |  | 0.034 | 1.071 | 0.024 |
| High UA |  |  |  |  |  |  | 0.040 | 1.085 | 0.008 |
| High FBG |  |  |  |  |  |  | 0.033 | 1.035 | 0.025 |
| Low HDL |  |  |  |  |  |  | 0.007 | 1.043 | 0.655 |
| R² | 0.401 | | | 0.403 | | | 0.465 | | |
| △R² | 0.401 | | | 0.002 | | | 0.062 | | |
| F | 288.422 | | | 248.975 | | | 172.540 | | |

Model 1 adjusted for age, BMI, smoking, drinking, and exercise; Model 2 further adjusted NAFLD based on Model 1; Model 3 further adjusted high TC, high TG, high UA, high FBG, and low HDL on the basis of Model 2
