# Supplementary material for: Association between non-alcoholic fatty liver disease and arterial stiffness measured by brachial-ankle pulse wave velocity: a cross-sectional population study
Source: PeerJ. 2025 May 19;13:e19405. doi: 10.7717/peerj.19405 (PMC12097236; doi:10.7717/peerj.19405)
Supplement: Supplemental Information 10 — Model 1 adjusted for age, BMI, smoking, drinking, and exercise; Model 2 further adjusted NAFLD based on Model 1; Model 3 further adjusted high TC, high TG, high UA, high FBG, and low HDL on the basis of Model 2. [file peerj-13-19405-s010.docx]

**Table S10**

**Multiple linear regression model after gender balance**

| **Characters** | **Model 1** | | | **Model 2** | | | **Model 3** | | |
| --- | --- | --- | --- | --- | --- | --- | --- | --- | --- |
|  | **β** | **VIF** | **P** | **β** | **VIF** | **P** | **β** | **VIF** | **P** |
| Male | 0.096 | 1.137 | ＜0.001 | 0.106 | 1.159 | ＜0.001 | 0.099 | 1.204 | ＜0.001 |
| Age | 0.643 | 1.007 | ＜0.001 | 0.638 | 1.013 | ＜0.001 | 0.548 | 1.178 | ＜0.001 |
| BMI | 0.067 | 1.046 | ＜0.001 | 0.039 | 1.204 | 0.003 | -0.002 | 1.254 | 0.895 |
| smoking | -0.022 | 1.119 | 0.083 | -0.023 | 1.120 | 0.065 | -0.028 | 1.127 | 0.018 |
| drinking | -0.022 | 1.076 | 0.033 | -0.028 | 1.076 | 0.023 | -0.036 | 1.093 | 0.002 |
| exercise | -0.157 | 1.005 | ＜0.001 | -0.149 | 1.018 | ＜0.001 | -0.125 | 1.039 | ＜0.001 |
| NAFLD |  |  |  | 0.077 | 1.188 | ＜0.001 | 0044 | 1.262 | ＜0.001 |
| Hypertension |  |  |  |  |  |  | 0.248 | 1.178 | ＜0.001 |
| High TC |  |  |  |  |  |  | 0.028 | 1.078 | 0.015 |
| High TG |  |  |  |  |  |  | 0.051 | 1.211 | ＜0.001 |
| High UA |  |  |  |  |  |  | 0.010 | 1.093 | 0.397 |
| High FBG |  |  |  |  |  |  | 0.042 | 1.072 | ＜0.001 |
| Low HDL |  |  |  |  |  |  | -0.008 | 1.049 | 0.477 |
| R² | 0.460 | | | 0.465 | | | 0.525 | | |
| △R² | 0.460 | | | 0.005 | | | 0.061 | | |
| F | 542.594 | | | 474.320 | | | 324.984 | | |

Model 1 adjusted for age, BMI, smoking, drinking, and exercise; Model 2 further adjusted NAFLD based on Model 1; Model 3 further adjusted high TC, high TG, high UA, high FBG, and low HDL on the basis of Model 2
