# Supplementary figures and images for: Association between non-alcoholic fatty liver disease and arterial stiffness measured by brachial-ankle pulse wave velocity: a cross-sectional population study
Source: PeerJ. 2025 May 19;13:e19405. doi: 10.7717/peerj.19405 (PMC12097236; doi:10.7717/peerj.19405)

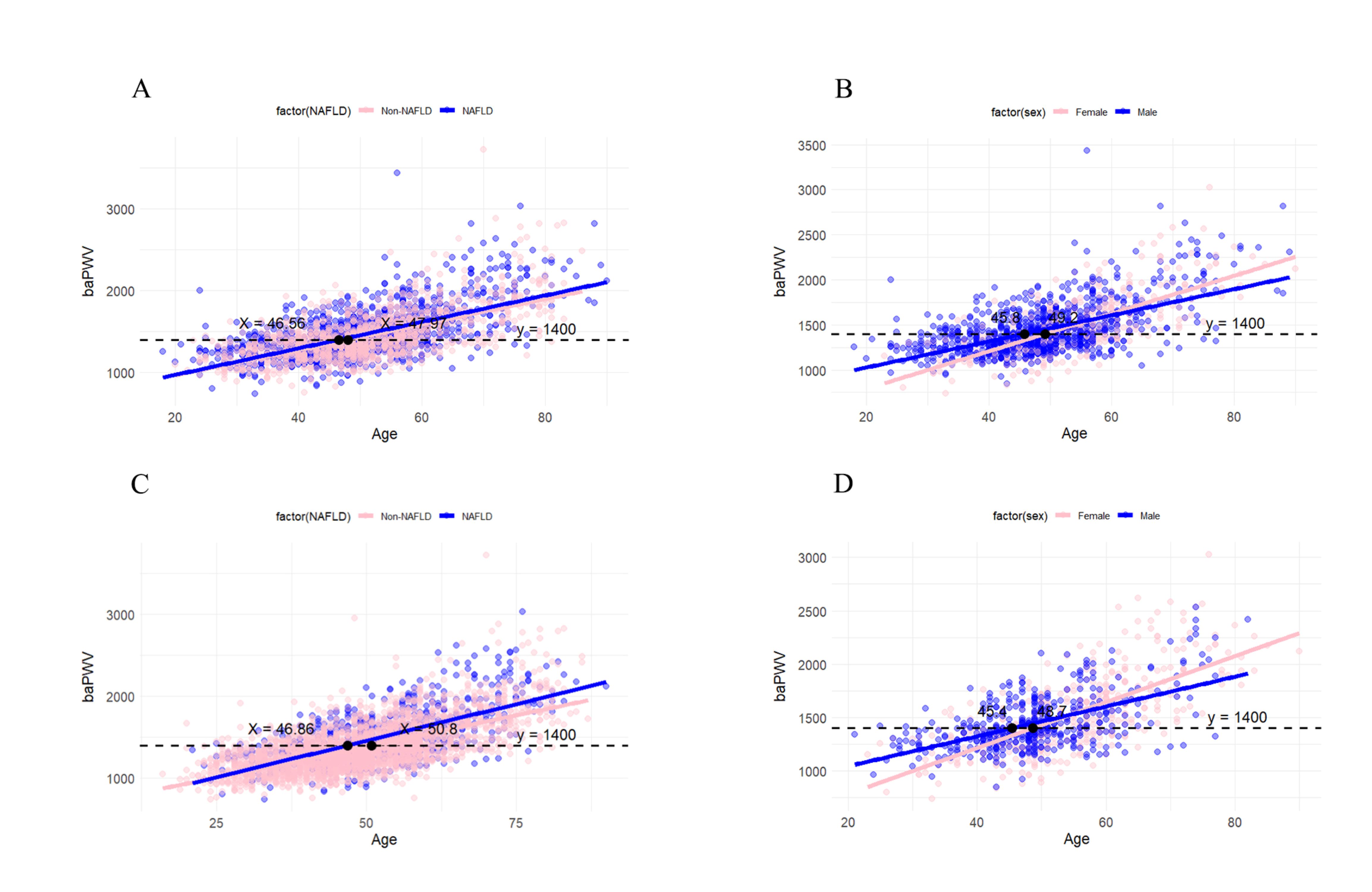

Supplement: Supplemental Information 11 — (A) the age-baPWV scatter plot of NAFLD and non-NAFLD groups after PSM. (B) the age-baPWV scatter plot of males and females in the NAFLD population after PSM; (C) the age-baPWV scatter plot of NAFLD and non-NAFLD groups in the sex-balanced subsample. (D) the age-baPWV scatter plot of males and females in the NAFLD population in the sex-balanced subsample. The number at the intersection of the black dashed line and the two fitted lines indicate the corresponding age when the baPWV reaches 1400 cm/s. [file peerj-13-19405-s011.jpg]
