# Supplementary material for: Association between non-alcoholic fatty liver disease and arterial stiffness measured by brachial-ankle pulse wave velocity: a cross-sectional population study
Source: PeerJ. 2025 May 19;13:e19405. doi: 10.7717/peerj.19405 (PMC12097236; doi:10.7717/peerj.19405)
Supplement: Supplemental Information 12 [file peerj-13-19405-s012.docx]

The determination of the biochemistry index is fulfilled by the central lab according to the uniform method of quality control. Each subject received venous blood drawn early in the morning after fasting for 10 hours to detect total cholesterol (TC), triglyceride (TG), high-density lipoprotein cholesterol (HDL-C), low-density lipoprotein cholesterol (LDL-C) and uric acid (UA) by the automatic biochemistry analyzer. Fasting blood glucose (FBG) is determined by the glucose oxidase method.

Height and weight are measured using an accurate standardized weight and height measuring instrument. Weight is accurate to 0.1kg, height is accurate to 0.1cm. Body mass index (BMI) was calculated by dividing body weight in kilograms by height in meters squared (kg/m2). According to the prevention and control guide of Chinese adults overweight and obesit([Zhou 2002](#_ENREF_1)), normal body weight, overweight, and obesity are defined as 18.5<BMI<23.9, 24≤BMI<27.9, and BMI≥28, respectively. Triglyceride (TG) ≥200mg/dL, total cholesterol (TC) ≥240mg/dL, high-density lipoprotein cholesterol (HDL-C) <40mg/dL, any of the three will be considered dyslipidemia([Zhu et al. 2018](#_ENREF_2)). Hypertension is diagnosed if blood pressure(BP) ≥140/90mmHg or if subjects have taken blood pressure medication in the last two weeks([Orimoloye et al. 2019](#_ENREF_3)). Fasting plasma glucose (FPG) ≥ 126mg/dL is considered hyperglycemia([Wang et al. 2019](#_ENREF_4)).

According to the World Health Organization, smokers are defined as those who have smoked continuously or cumulatively for six months or more in their lifetime([World Health 1998](#_ENREF_5)). According to the World Health Organization, the following is the definition of physical activity: people aged 5-17 years old average at least 60 minutes per day per week of moderate to vigorous intensity physical activity, mainly aerobic exercise, at least 3 days per week of vigorous intensity aerobic exercise; People aged 65 years and older do 150-300 minutes of moderate-intensity aerobic activity or 75-150 minutes of vigorous-intensity aerobic activity per day, or an equivalent combination of these moderate-intensity and vigorous-intensity activities over the course of a week, and do muscle-strengthening activities at least 2 days per week([World Health 2020](#_ENREF_6)). Drinking is defined as a history of alcohol consumption in the last six months, and the amount of alcohol consumed is consistent with no more than 140g per week for men and no more than 70g per week for women([World Health 1994](#_ENREF_7)).

REFERENCE:

Zhou BF. 2002. Predictive values of body mass index and waist circumference for risk factors of certain related diseases in Chinese adults--study on optimal cut-off points of body mass index and waist circumference in Chinese adults. *Biomed Environ Sci* 15:83-96.

Zhu JR, Gao RL, Zhao SP, Guoping LU, Zhao D, Jianjun LI, Zhu JR, Gao RL, Zhao SP, and Guoping LU. 2018. 2016 Chinese guidelines for the management of dyslipidemia in adults. *Journal of Geriatric Cardiology Jgc* 15:1-29.

Orimoloye OA, Mirbolouk M, Uddin SMI, Dardari ZA, and Blaha MJ. 2019. Association Between Self-rated Health, Coronary Artery Calcium Scores, and Atherosclerotic Cardiovascular Disease Risk: The Multi-Ethnic Study of Atherosclerosis (MESA). *JAMA Network Open* 2:e188023.

Wang L, Niu JY, Zhao ZY, Li M, Xu M, Lu JL, Wang TG, Chen YH, Wang SY, Dai M, Li L, Liu SS, Wang WQ, Xu Y, and Bi YF. 2019. Ideal Cardiovascular Health is Inversely Associated with Subclinical Atherosclerosis: A Prospective Analysis. *Biomed Environ Sci* 32:260-271. 10.3967/bes2019.036

World Health O. 1998. Guidelines for controlling and monitoring the tobacco epidemic. Geneva: World Health Organization.

World Health O. 2020. *WHO guidelines on physical activity and sedentary behaviour*. Geneva: World Health Organization.

World Health O. 1994. Lexicon of alcohol and drug terms. Geneva: World Health Organization.
